# T1 measurements identify extracellular volume expansion in a genotyped hypertrophic cardiomyopathy population with and without left ventricular hypertrophy

**DOI:** 10.1186/1532-429X-15-S1-O21

**Published:** 2013-01-30

**Authors:** Siddique Abbasi, Ravi Shah, Tomas G Neilan, Bobby Heydari, Yucheng Chen, Michael Jerosch-Herold, Raymond Y Kwong, Carolyn Ho

**Affiliations:** 1 Department of Cardiovascular Medicine, Brigham and Women's Hospital/Harvard Medical School, Boston, MA, USA; 2 Cardiology, Massachusetts General Hospital, Boston, MA, USA

## Background

Myocardial fibrosis is a hallmark of hypertrophic cardiomyopathy (HCM) and may contribute to arrhythmias and heart failure. Sarcomere mutations appear to induce profibrotic changes before left ventricular hypertrophy (LVH) develops. Measuring T1 relaxation times with contrast cardiac magnetic resonance (CMR) allows robust quantification of the cardiac extracellular volume (ECV) and non-invasive assessment of diffuse myocardial fibrosis.

## Methods

A genotyped HCM population underwent contrast CMR with measurement of T1. Subjects included sarcomere mutation carriers with LVH (G+/LVH+, n = 37) and without LVH (G+/LVH-, n = 30); HCM patients without mutations (sarcomere-negative HCM, n = 11); and mutation-negative healthy controls (n = 10). Concurrent echocardiography and serum biomarkers of collagen synthesis, hemodynamic stress, and myocardial injury were available in a subset.

## Results

Late gadolinium enhancement (LGE) was present in >60% of overt HCM patients but absent from G+/LVH- subjects. Compared to controls, ECV was increased in patients with overt HCM, as well as G+/LVH- mutation carriers (ECV, 0.37±0.01, 0.33±0.01, 0.26±0.01 in G+/LVH+, G+/LVH-, controls, respectively, P<0.001). ECV was correlated with NT-proBNP levels (r=0.61, P<0.001) and global E' velocity (r=-0.48, P<0.001). ECV and LGE were higher in sarcomeric HCM than sarcomere-negative HCM.

## Conclusions

Myocardial ECV is increased not only in sarcomere mutation carriers with overt HCM, but also those with normal LV wall thickness. These data provide further evidence that fibrotic remodeling is triggered early in disease pathogenesis. Assessing ECV may help characterize the development of HCM-induced myocardial fibrosis, ultimately supporting novel strategies to modify disease by targeting interstitial fibrosis.

## Funding

This work was supported by grants from the National Institutes of Health (CYH and RYK).

**Table 1:** CMR Metrics of Fibrosis and Serum PICP levels

	Control N=10	p-value* Control vs Preclinical	Preclinical HCM N=30	p-value* Preclinical vs Overt	Overt HCM N=37	p-value* Control vs Overt
ECV (range)	0.26 ± 0.01 (0.24-0.31)	<0.001	0.33 ± 0.01 (0.23-0.38)	<0.001	0.37 ± 0.01 (0.31-0.51)	<0.001

ECV, excluding LGE	0.26 ± 0.01	<0.001	0.33 ± 0.01	<0.001	0.37 ± 0.01	<0.001

LGE present, % (#yes / #no)	0% (0/10)		0% (0/29)		78.4% (29/8)	

LGE, g	0	0.8	0	0.004	16.7 ± 4.9	0.003

LGE, % LV mass	0	0.7	0	0.0006	7.76 ± 1.96 (0.9-44.6%)	0.001

PICP, μg/L	63.08 ± 4.19	<0.001	83.81 ± 4.39	0.035	108.04 ± 9.81	<0.001

Data are presented as means adjusted for family relations and age ± standard error *p values <0.017 are statistically significant and adjusted for age and family relations ECV = extracellular volume; LGE = late gadolinium enhancement; PICP = carboxy-terminal propeptide of procollagen type I

**Figure 1:**
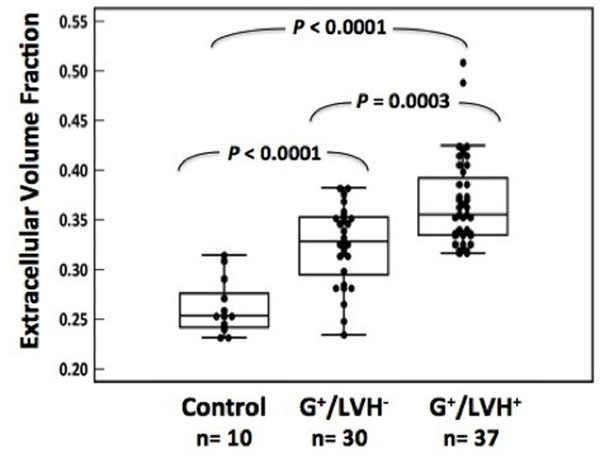
ECV is significantly increased in HCM sarcomere mutation carriers both with and without LVH Compared to normal controls, ECV was 25% in G+/LVH- subjects and 42% higher in G+/LVH+ HCM patients (p<0.001 for all comparisons to controls). ECV was further increased by 13% in overt HCM compared to G+/LVH- subjects (p=0.0003).

